# The Cell Nucleus and Aging: Tantalizing Clues and Hopeful Promises

**DOI:** 10.1371/journal.pbio.0030395

**Published:** 2005-11-15

**Authors:** Paola Scaffidi, Leslie Gordon, Tom Misteli

## Abstract

Recent evidence links structural proteins in the cell nucleus with aging.

There are a handful of biological questions that affect all of us directly in everyday life. How are emotions formed, what is the basis for consciousness, and why do we look the way we do? One that strikes particularly close to home is the question of how we age. The sheer complexity of this problem has had many scientists throw up their hands in frustration and most of the postulated theories have been vague and generally have involved ill-defined wear-and-tear mechanisms. But the pursuit of the biological basis of aging has been revitalized within the last decade by studies in yeast, worms, flies, and mice that have firmly established that there indeed exist specific molecular mechanisms that contribute to the aging process [1]. These efforts point to several distinct, likely interrelated, mechanisms, ranging from improper protein metabolism, to alterations of specific signaling pathways, progressive damage due to generation of oxidative free radicals, and increased genome instability.

Although much has been learned about the aging process from simple model organisms, one intuitively suspects that things might be somewhat different when it comes to human aging. So how does one best study the molecular basis of human aging? The answer might be premature aging diseases, or progeroid syndromes. The advantage of these often rare diseases is that they are mostly monogenic and thus experimentally tractable. On the other hand, one should keep in mind that such disorders usually only mimic some of the features of normal aging and it can be difficult to distinguish true aging symptoms from unrelated developmental defects. Regardless, it appears that progeroid syndromes may be legitimately used as model systems to investigate the physiological processes contributing to aging. In fact, the study of some of these diseases has recently brought tantalizing clues as to how we age. One of the most intriguing ones is a possible involvement of structural components of the cell nucleus [2].

## Aging and the Cell Nucleus

The cell nucleus in higher organisms is now recognized as a complex, highly organized repository of an individual's genetic information. The typical nucleus contains distinct functional neighborhoods made up by nonrandomly positioned chromosomes and proteinaceous subcompartments in which specific processes, including gene expression, occur [3]. Which molecular mechanisms establish and maintain the structural integrity of the nucleus is largely unknown and represents one of the most exciting fields in modern cell biology. Having said that, one of the major structural elements of the nucleus, the nuclear lamina, has been known and studied for decades [2]. The lamina is made up of A-, and B-type lamins, which are intermediate filament proteins that form an interwoven network situated at the very periphery of the nucleus underlying the nuclear membrane. This structure has long been thought to act as a shield to protect the genome from mechanical stress. More recently, this architectural feature of the nucleus has also been recognized as potentially playing a regulatory role in gene expression because lamins interact with chromatin and might serve to anchor and organize genome regions in space [2].

The first hint to a surprising connection between nuclear architecture and aging came from yeast, when Leonard Guarente and colleagues found that a protein, Sir4, whose mutation results in extension of life span, localizes to the nucleolus, one of the most prominent subcompartments of the cell nucleus [4]. The link between aging and nuclear organization was further strengthened by the observation that the localization of the protein and the morphology of the nucleolus itself changed as yeast cells aged. However puzzling and provocative these observations were, it was unclear whether these structural reorganizations were a cause or consequence of aging, and it seemed a stretch to imagine that the same mechanisms might apply to human cells.

## Hutchinson-Gilford Progeria Syndrome

**Figure 1:**
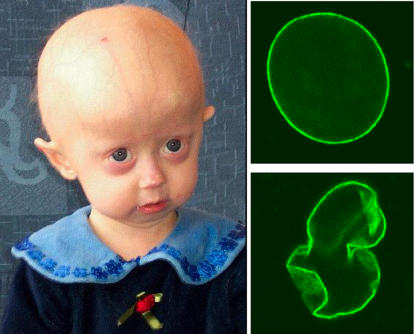
Hutchinson-Gilford Progeria Syndrome HGPS is a childhood disorder caused by mutations in one of the major architectural proteins of the cell nucleus. In HGPS patients the cell nucleus has dramatically aberrant morphology (bottom, right) rather than the uniform shape typically found in healthy individuals (top, right).

The definitive proof for a causal connection between nuclear architecture and human aging came with a stunning discovery in the summer of 2003, when the groups of Francis Collins and Nicolas Levy identified mutations in the lamin A gene *(LMNA)* as the genetic cause of the segmental premature aging disease Hutchinson-Gilford progeria syndrome (HGPS) [5,6] (Box 1). Children with HGPS usually experience normal fetal and early postnatal development but die of severe atherosclerosis at an average age of 13 years [7]. The initial physical signs of HGPS include severe failure to thrive, heralding severe lipoatrophy, bony abnormalities, a small, beaked nose and receding mandible, complete hair loss, and speckled hypopigmentation with some areas of tight, hard skin. As the disease progresses, vascular plaques become pervasive, leading to strokes and heart attacks. In short, these children give the distinct physical impression of being many decades older that they really are. However, HGPS children are neurologically unaffected, so that their emotional and developmental stages are age-appropriate. A six-year-old with HGPS may look physically like an old person, but is ready to enter first grade along with the rest of his or her peers (Figure 1). In a sense, for families and friends, having a child whose mind and personality progress normally is a great fortune. In another sense, watching a child whose mind is so full of promise and joy experience angina, strokes, and heart attacks that are usually reserved for the elderly is devastating.

**Box 1. The Cell Biology of HGPS**

In more than 80% of cases the gene defect responsible for HGPS is a single spontaneous mutation in codon 608 of the *LMNA* gene, which encodes both lamin A and lamin C [6]. This base change produces a silent amino acid mutation and would therefore have no consequences for the lamin A protein. Unfortunately, the mutation activates a cryptic splice site in *LMNA*, resulting in aberrant removal of a part of the *LMNA* RNA during the RNA splicing reaction and generation of a protein lacking 50 amino acids towards its C-terminal end. This mutant protein is referred to as progerin. The progerin protein appears to act in a dominant negative fashion, and although its modus operandi is unclear at the moment, its action seems to be related to its extensive post-translational modifications. Normal lamin A is produced as a pre-lamin A protein that undergoes a complex set of modifications starting with carboxymethylation, followed by cleavage of the terminal three amino acids, farnesylation at the C-terminus, and subsequent proteolysis of its terminal 15 amino acids, leading to the removal of the farnesyl group. Although it can be farnesylated, pre-progerin lacks the endoprotease recognition site necessary for executing the final cleavage step and thus accumulates in a farnesylated form [19]. Since the farnesyl group is important for the protein's localization to the nuclear periphery, progerin accumulates in the lamina and this is presumably where it exerts its negative functional effects. Consistent with that notion, loss of the protease responsible for the cleavage event leads to premature aging in mice and humans [20,21]. Given the critical importance of the lamins in nuclear architecture, it is not surprising that the cells of HGPS patient are characterized by dramatic aberrations in nuclear architecture, particularly changes in the formation of their shape, loss of internal heterochromatin, and changes in the distribution of numerous nuclear proteins [14,22].

**Figure:**
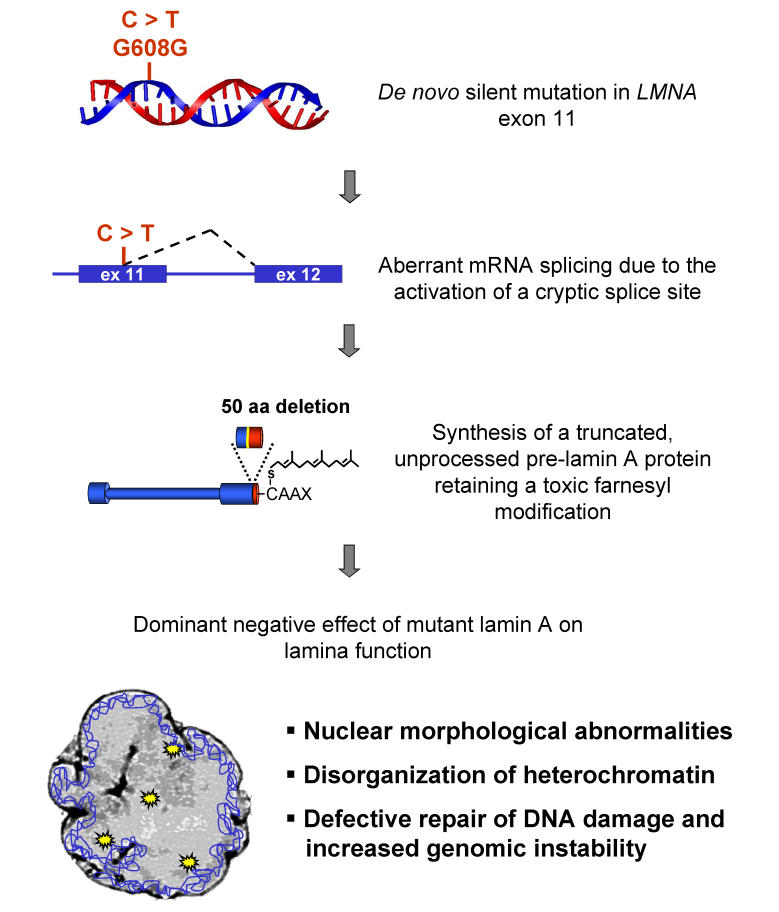
The Molecular Basis of Nuclear Defects in HGPS

The involvement of lamin A in this disease was initially puzzling. It is not apparent how a protein whose function is to maintain proper nuclear architecture may cause premature aging. Two hypotheses rapidly emerged based on what cell biologists had learned about this intensely studied protein in the last two decades. It could be that the mutant lamin A protein weakens the nuclear lamina and in that way reduces the resistance of the cells of HGPS patients to the types of mechanical stress encountered in the body, heralding cellular dysfunction and death. This explanation makes much sense since many of the primarily affected tissues, such as skin and vasculature, are under intense mechanical stress. On the other hand, recent work showing that lamins directly and indirectly interact with chromatin point to the possibility that changes in the lamina might lead to misregulation of sets of genes in HGPS patients. Although plausible, it is not obvious how the gene misregulation model can account for the aging effect seen in patients, particularly since it has so far proven difficult to identify sets of genes that are commonly misregulated in these patients. Even the mechanical stress model is not completely satisfying since it is based on one of those ill-defined wear-and-tear scenarios so often invoked in the aging process. The distinct possibility that aspects of both models might apply is suggested by the fact that several genes that respond to mechanical signal transduction via the cytoskeleton are affected in cells lacking lamin A [8].

A possible breakthrough in understanding how a structural protein of the cell nucleus may be a key player in aging came just a few months ago. Zhang and colleagues discovered that cells in HGPS patients have increased genomic instability and DNA damage and that the DNA repair machinery does not function as effectively as it does in healthy individuals [9]. In particular, it appears that in the cells of individuals with HGPS, the repair machinery is not as efficiently recruited to sites of damage, and, consequently, DNA breaks are less efficiently repaired. Although unproven at this point, one can imagine that defects in the lamina structure could prevent the efficient capture of repair factors [9,10]. Admittedly, increased genome instability must also be considered a wear-and-tear mechanism, but it deserves more credence than some of the other mechanisms because genome instability is a feature shared amongst virtually all premature aging disorders.

## Genomic Instability and Aging

The clearest link between genomic instability and accelerated aging comes from a different progeroid disease, Werner syndrome (WS). This is an autosomal recessive disorder resulting from loss of function of a DNA helicase, the WRN RecQ helicase [11]. Compared to HGPS, this condition has a later onset, with patients developing normally until puberty and showing aging symptoms in early adulthood. Death generally occurs in the fifth decade of life, mainly because of cancer and cardiovascular disease. Extensive research over the past decade has shed light on the molecular and cellular defects underlying the disease phenotype, making WS a perfect example of how important cell biology can be in unraveling mechanisms of premature aging. The WRN protein is involved in DNA replication and recombination and its main function is to reinitiate stalled replication forks. In the cells of WS patients, the absence of the WRN protein results in defective replication, inefficient DNA repair, and chromosome rearrangements. Thus, altered DNA metabolism in WS cells directly causes defective maintenance of genome integrity, and this, in turn, results in cancer predisposition in patients [11].

The complex nature of the interplay between nuclear architecture, DNA metabolism, and aging becomes clear when considering a subset of patients who were diagnosed with WS based on their clinical symptoms, but, puzzlingly, do not carry a mutation in the WRN gene—instead, they have mutations in *LMNA* [12]. Clearly, the fact that WS symptoms can be recapitulated by mutations in a gene that gives rise to another premature aging disease is hardly coincidental. This remarkable confluence of observations reinforces the idea that HGPS and WS might share a similar underlying molecular mechanism, possibly genomic instability.

Accelerated aging, however, probably does not involve only genomic instability. WS has also been critical in revealing a potential role of cellular senescence in the aging process [13]. Cells entering senescence, i.e., permanent arrest of cell division, undergo phenotypic changes and display several functional abnormalities compared to their proliferating counterparts. A second hallmark of cells in WS patients is short replicative life span in culture, due to telomere dysfunction. Although the molecular details of how the WRN protein affects telomere metabolism are not fully understood, the picture emerging is that accumulation of senescent, dysfunctional cells in WS patients might compromise the effectiveness of tissues and organs, leading to accelerated aging. Extending this model, cellular senescence has been postulated to also play a key role in physiological aging.

These observations on WS and the occurrence of genomic instability in premature aging diseases crystallize a tantalizing antagonism between aging and cancer [13]. That these two are linked is clear from the fact that age is the single largest risk factor for the development of cancer. In addition, genomic instability as seen during aging is a hallmark of cancer cells. Paradoxically, what we have learned from WS suggests that increased senescence appears to lead to premature aging, but on the other hand, cellular senescence is now also recognized as a defense mechanism to effectively stop potentially harmful, cancer-forming cells from proliferating. Clearly, organisms must find a fine balance between these opposing effects. How this equilibrium is achieved during the physiological aging process is one of the fascinating mysteries of aging.

## Finding the Fountain of Youth

One of the lures of aging research is of course to find a fountain of youth—an elixir that prolongs life. Although several commercial entities are unleashing their resources at this tempting goal, given the inconclusive nature of most molecular aging pathways currently under consideration, these efforts seem unlikely to come to fruition in the near future. However, therapeutic intervention may be realistic for premature aging disorders as their molecular basis is becoming well defined. The feasibility of anti-aging intervention is made clear in the case of HGPS. Since the discovery of its genetic cause less than two years ago, at least two fascinating, yet realistic, strategies of molecular treatment have been opened. The first is based on correcting the primary defect at the level of pre-mRNA. This strategy involves correcting the aberrant splicing event caused by the HGPS mutation (Box 1). Proof of principle that this is possible has recently been provided by introduction of small oligonucleotides specifically targeted to the HGPS splice site, preventing its use and restoring normal splicing and cellular behavior [14]. If these results from cell culture experiments can be extended to organisms, they might provide a promising avenue of intervention. A second potential therapeutic approach for HGPS is founded in the complex post-translational processing of the lamin A protein by farnesylation (Box 1). Inhibitors of the farnesylation reaction, so called farnesyl transferase inhibitors, have recently been developed as potential cancer therapeutics and might also be effective in HGPS. The hope would be that these inhibitors will decrease the amount of harmful farnesylated pre-progerin in the cell and thus alleviate cellular HGPS symptoms. Fortunately, ongoing clinical trials on farnesyl transferase inhibitors show little toxicity, and early studies in cell culture have already shown a reversal of some of the cellular defects associated with HGPS [15–18].

**Figure 2:**
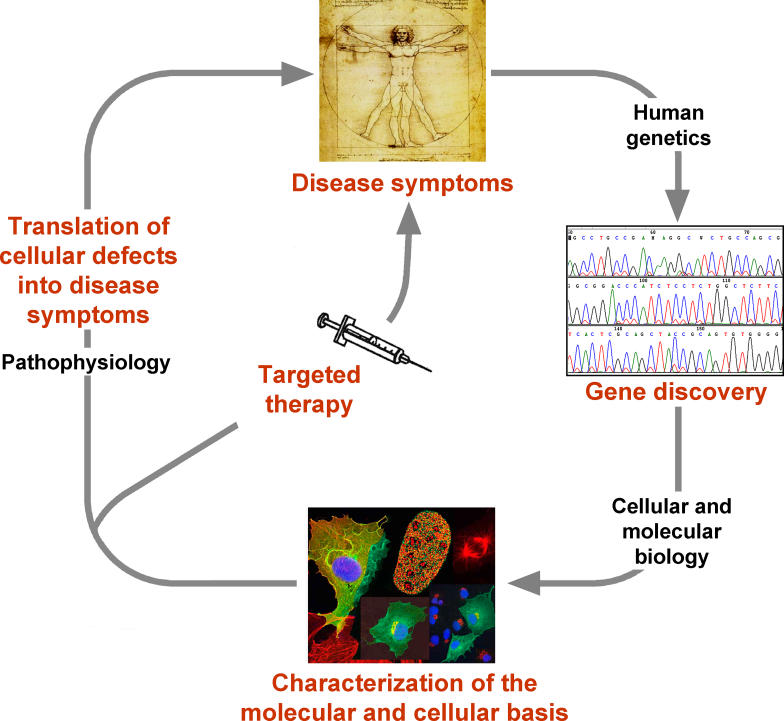
The Disease–Discovery–Therapy Cycle Diseases are clinically defined by their symptoms. The genetic basis of disease is revealed by gene discovery. The cellular and molecular basis of a disease is characterized by cell biological approaches. These in turn provide the foundation to develop targeted therapies to alleviate symptoms and to fully understand the pathophysiology of the organismal disease symptoms.

The story of HGPS is an impressive example of the interplay of basic and clinical science. It is also a showcase for how modern biology deals with disease (Figure 2). Within less than two years, we have gone from only knowing the symptoms of the disease to identifying the disease-causing gene and learning much about how the mutant protein behaves in patient cells. The challenge ahead is to close the circle and to apply what we have learned about the cell biology of this disease to the development of therapeutic approaches. The insights from HGPS have also opened an entirely new and fascinating vista on the aging process. Who would have guessed two years ago that architectural elements of the cell nucleus might contribute to aging? These advances have put us in the rare and desirable situation of possibly being able to making significant steps towards understanding one of the most fascinating problems in biology—and at the same time do some good for patients and their families.

## Abbreviations

HGPS: Hutchinson-Gilford progeria syndrome
*LMNA*: lamin A gene
